# Comparison of the effects of spark plasma at two different voltages on aspects influencing skin rejuvenation

**DOI:** 10.1371/journal.pone.0333006

**Published:** 2025-10-08

**Authors:** Erfan Ghasemi, Mohammad Ali Nilforoushzadeh, Mohammadreza Khani, Mohammad Amir Amirkhani, Maryam Nouri, Parisa Charipoor, Babak Shokri

**Affiliations:** 1 Laser and Plasma Research Institute, Shahid Beheshti University, Tehran, Iran; 2 Skin and Stem Cell Research Center, Tehran University of Medical Sciences, Tehran, Iran; 3 Skin Repair Research Center, Jordan Dermatology and Hair Transplantation Center, Tehran, Iran; 4 Physics Department of Shahid Beheshti University, Tehran, Iran; Shiraz University of Medical Sciences, IRAN, ISLAMIC REPUBLIC OF

## Abstract

Skin is not only the body’s largest organ but also one of the most noticeable and hard-working parts. Every day, it shields us from things like bumps, changes in temperature, and harmful substances in the environment. But as we age, this protection starts to weaken. Spending too much time in the sun, being exposed to pollution, and other stressors lead to visible signs of aging, such as dry skin, uneven color, fine lines, and a loss of firmness and elasticity. Recently, spark plasma has shown great potential as a gentle and non-invasive way to help the skin heal itself. In earlier research [16], we studied how applying spark plasma at 3.44 kV affected the skin, and the results were very encouraging. Inspired by those findings, we continued with more research. In this study, we tested two different levels of spark plasma—3.44 kV and 4.88 kV—to see how each affected the skin in different ways. We measured changes in skin color, firmness, density, tightness, moisture, and thickness using both MPA9 biophysical tests and ultrasound imaging. We noticed that both levels led to visible improvements in the skin, but the higher voltage had a stronger effect, especially when it came to increasing collagen and speeding up the healing process. Interestingly, there was a brief increase in redness and melanin right after the treatments, but these effects went away and returned to normal within four weeks. Most importantly, there were no scars, long-term irritation, or harmful side effects during or after the treatments.

## Introduction

Skin rejuvenation is one of the most popular cosmetic procedures worldwide, aimed at addressing age-related changes such as wrinkles, laxity, and uneven pigmentation. While traditional methods such as ablative fractional CO₂ lasers have shown clinical efficacy, their use is often limited by downtime and thermal injury risks [1]. In contrast, plasma-based technologies are gaining attention for their non-ablative, tissue-preserving effects. [2]. Plasma, often described as the fourth state of matter, is made by applying high-voltage electricity to gas molecules. This creates a type of gas that’s only partly ionized and includes ions, electrons, and active oxygen and nitrogen molecules [3]. These active substances form a medium that can react with living tissues, which is the basis for the field of plasma medicine [3].

Several studies have demonstrated the therapeutic potential of plasma in dermatological applications. Scarano and their team found that using voltaic arc resurfacing can remove small lines, especially around the mouth, and also make deeper lines look better by tightening the skin [4]. In another study, the same group noticed that there was no damage to the deeper layers of the skin and that the skin healed properly without any dead tissue, which suggests that the treatment works mainly on the surface without causing harm [5,6].

The skin serves as the body’s largest organ and primary protective barrier against environmental insults. It comprises three layers: the epidermis, dermis, and hypodermis. The epidermis is predominantly made up of keratinocytes and melanocytes, while the dermis houses fibroblasts, collagen, elastin, vasculature, and hair follicles [7,8]. Key parameters used to assess skin health and aging include thickness, elasticity, transepidermal water loss (TEWL), melanin content, and structural protein integrity [9]. Melanocytes contribute to pigmentation by synthesizing and transferring melanin to keratinocytes, where it localizes above the nucleus to protect DNA from UV damage [10]. Meanwhile, TEWL is a commonly used marker for evaluating the integrity of the skin barrier, particularly in conditions such as eczema, irritation, or post-treatment barrier disruption [11,12].

Structural proteins like collagen and elastin play key roles in maintaining skin strength and elasticity. Aging is associated with degradation or disorganization of these proteins, leading to wrinkles, rough texture, uneven pigmentation, and loss of firmness [13]. Previous research using plasma jets has shown reduced hydration in superficial layers, while confocal laser imaging has demonstrated improved collagen structure post-treatment [14,15].

Our earlier study [16] demonstrated that spark plasma applied at 3.44 kV significantly improved several skin characteristics in animal models. However, the biological impact of increasing the plasma voltage has not been fully explored. This study investigates the voltage-dependent effects of spark plasma at two intensities (3.44 and 4.88 kV) on biophysical and structural properties of rat skin. It checks several things like how elastic the skin is, how much water it loses through the surface, skin color changes due to melanin and redness, how thick and dense the tissue is, and the structure seen under a microscope. By comparing the results from the two voltage levels, the study wants to better understand how effective and safe spark plasma is at different energy levels, which helps in testing it for possible use in future skin treatments.

## Materials and methods

### Animal models and research groups

In this experiment, male Wistar rats (4 months old) weighing 250 ± 50 gr were obtained from the Pasteur Institute (Tehran, Iran). The 18 rats were divided into three groups of six (2 treatment groups and 1 control group) and kept in separate cages with easy access to water and food under standard laboratory settings (room temperature, atmospheric pressure, humidity 30 ± 10%, light/dark cycle 12 hours). Following the experiment, all rats were donated to the Faculty of Biology at Shahid Beheshti University for survival. The Tehran University of Medical Sciences ethics committee.

### Plasma device

**Fig 1:**
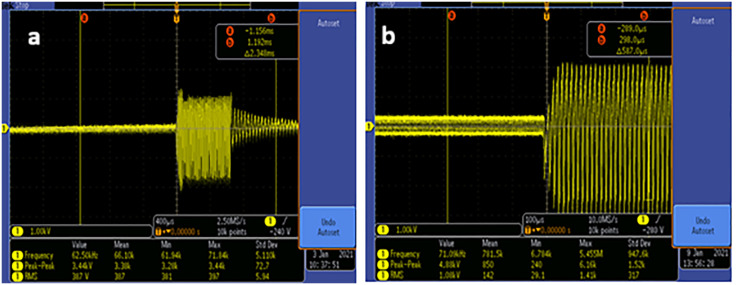
Cold plasma device characteristics: voltage and frequency (a) 3.44 kV [16] (b) 4.88 kV [Representative oscilloscope measurements; no statistical analysis applied].

In this study, spark plasma from Plasma Fanavar Jam Engineering Company(Plasma Beauty 100) manufactured in Tehran, Iran, was employed in two modes. The power of the Plasma Beauty 100 gadget ranges from 10% to 100% at a distance of 10. For this study, two settings were used: 50%, representing half of the device’s full capacity, and 100%, reflecting maximum power output. The electrical characteristics of the plasma device in these two modes were examined and demonstrated using an oscilloscope and a high-voltage probe (Tekterorix, P6015A, 1:1000). The voltage waveforms were sinusoidal, as shown in Fig 1a, 1b). In medium mode, the device operates with a peak-to-peak voltage of 3.44 kV and a frequency of 62.5 kHz (Fig 1a), while in high mode, the peak-to-peak voltage is 4.88 kV, with a frequency of 71.09 kHz (Fig 1b). In the current SparkMED system, voltage and frequency are interlinked, with higher voltages resulting in slightly elevated frequencies. Due to the limitation of utilizing animals, the study was compared in two modes of maximum power and medium power, so that the operator can finally select the preferred mode based on demand.

**Fig 2:**
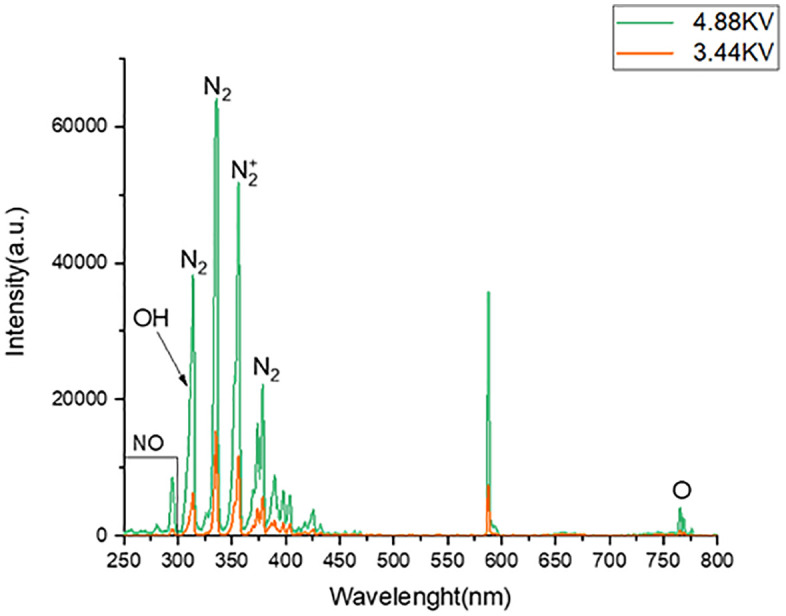
Characteristics of the cold plasma device: spectrum analysis and the excited species created by the device [Representative OES data; no statistical analysis applied].

Optical emission spectroscopy (OES; Avaspec3648USB2) was used to examine reactive species produced in the plasma at wavelengths ranging from 250 to 800 nm. As seen in Fig 2 [17], detected emissions included the N₂/N₂ ⁺ bands (315–380 nm), OH (309 nm), and NO (297 nm). [17].

**Fig 3:**
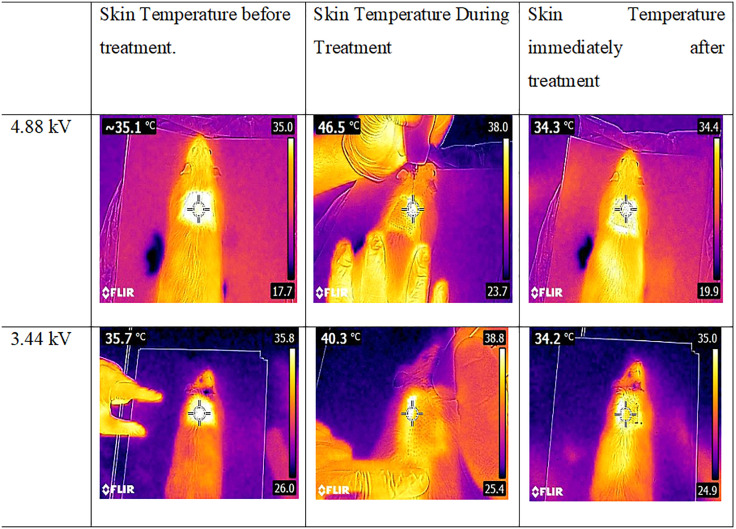
Rat skin temperature changes: before, after, and during treatment.

The skin surface temperature during treatment was monitored using an infrared thermal camera (FLIR-E63900-T198547, Estonia; thermal sensitivity <0.06°C). Plasma exposures were evaluated at both voltage levels to assess their thermal safety profiles (Fig 3 and Table 1).

**Table 1:** Skin Temperature Data.

Voltage	Before Treatment (°C)	During Treatment (°C)	immediately Treatment (°C)
3.44 kV	35.7	40.3	34.2
4.88 kV	35.1	46.5	34.3

### Evaluation of biophysical skin measurements

Skin biophysical parameters—including melanin index, erythema index, transepidermal water loss (TEWL), elasticity, and skin thickness—were quantitatively measured before treatment, immediately after, and at 2 and 4weeks post-treatment.

Melanin and erythema indices were measured using the Mexameter® MX 18 (Courage + Khazaka Electronics, Cologne, Germany), which operates based on reflectance spectrophotometry. The device emits green (568 nm), red (660 nm), and infrared (870 nm) wavelengths, and quantifies absorption by skin chromophores to determine pigmentation and vascularity levels.

TEWL was assessed using the Tewameter® TM 300 probe (Courage + Khazaka), a non-invasive system that measures the water vapor flux density gradient from the skin surface to evaluate barrier integrity. Measurements were taken under controlled conditions (23°C, 40% humidity) to ensure consistency.

Skin elasticity was evaluated using the Cutometer® Dual MPA 580 (Courage + Khazaka GmbH, Germany), which utilizes negative pressure to deform the skin and measure its viscoelastic response. Two parameters were recorded:

R2: overall elasticity (ratio of total recovery to total deformation)

R5: net elastic recovery (ratio of immediate recovery to deformation)

Skin thickness and structural density were analyzed using high-frequency ultrasound imaging (DUB SkinScanner Taberna pro, Germany) equipped with a 75 MHz probe. This allowed visualization of skin layers up to 1 mm depth, effectively capturing both epidermis and dermis. The hypodermis could not be accurately assessed. All scans were performed on the interscapular region, with hair shaved and probe placement standardized to reduce variability.

All measurements were repeated at consistent anatomical sites and conducted under standardized environmental conditions (23°C, 40% humidity) with the animals in a relaxed state.

### Treatment and preparation

Eighteen Wistar rats were split into three groups randomly, with six rats in each group.

Group 1 got plasma treatment at 3.44 kV.

Group 2 got plasma treatment at 4.88 kV.

Group 3 was the control group and didn’t get any treatment.

**Fig 4:**
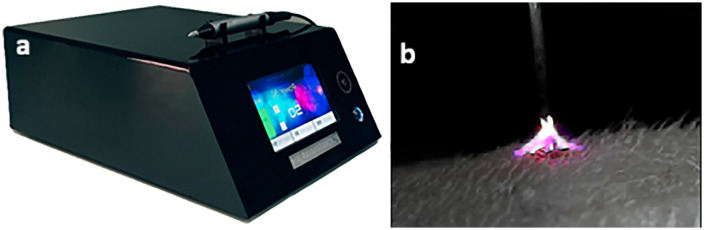
High-voltage spark plasma device and plasma formation at the skin interface. **a)** Spark plasma device, **b)** The plasma formed at a voltage of 3.44 kV between the needle and the skin [16] (Fig 4b was captured using the HiView microscope to illustrate the plasma as it contacts the skin.).

All animals were given intraperitoneal injections of xylazine hydrochloride (10 mg/kg) and ketamine hydrochloride (100 mg/kg) to induce anesthesia prior to any treatment. The treatment area was chosen as the back of the neck, which is easy to reach and has a similar structure in all rats. The area was shaved and cleaned, and a cream called Xyla-P was applied for 20 minutes to numb the skin. Then, plasma was applied using a device called SparkMED (made by Jam Technology in Iran) in a triangular pattern (Fig 4a, 4b). Each treatment lasted just one second, and each rat only had one session. Right after the treatment, the treated area was measured with a ruler to check for any visible shrinking. Skin measurements were taken at four different times: before the treatment, right after, and again at 2 weeks and 4 weeks later. The measurements included R2, R5 (which shows how elastic the skin is), TEWL (how much moisture escapes), melanin (skin pigment), and erythema (redness). Each of these was checked three times on each rat, and the average was used for the analysis. The control group had the same checks done, but they didn’t receive any treatment.

### Tissue sampling and histological analysis

A separate set of eighteen Wistar rats (6 rats per group) was used exclusively for histological analysis and was completely independent of the animals used for biophysical measurements. Each rat underwent two skin biopsies: one before and one four weeks after plasma treatment.

In the first stage, a 4 mm punch biopsy was taken from the shaved interscapular region (upper back) of each rat under general anesthesia. The biopsy site was then allowed to fully heal over a 10-day period, ensuring complete epithelial regeneration.

After recovery, spark plasma was applied directly to the healed area using the SparkMED device at one of two voltage levels—either 3.44 kV or 4.88 kV—depending on the assigned group. A separate control group followed the same biopsy and healing schedule but did not receive any plasma treatment.

Four weeks after plasma exposure, a second biopsy was taken from the exact same area as the first. Both pre- and post-treatment tissue samples were fixed in 10% formalin for histological processing. To ensure consistency, all biopsies were taken at the same depth, location, and under identical conditions.

The collected tissue samples were stained using hematoxylin and eosin (H&E) and Masson’s trichrome methods. Microscopic examination was carried out using a KF2 optical microscope (ZEISS West Germany®) equipped with calibrated lenses (Erma Japan®) and a Sony Cybershot camera (magnification 4 × 10 × 40). Quantitative measurements—including epidermal thickness, collagen fiber thickness, and collagen area—were performed using ImageJ software in combination with Carl Zeiss Axiovision Version 4.8 (refer to Fig 9 and Table 4).

**Table 4:** Changes in rats’ skin average epithelium (µm) and collagen (µm) over 4 weeks following plasma processing.

	Average epithelium (μm)			Average collagen (μm)		
	Before	After 4 weeks	The percentage of modifications	Before	After 4 weeks	The percentage of modifications
4.88 kV	26.46	43.86	65.75%	4.015	4.685	16.68%
3.44 kV	32.84	35.86	9.19%	4.270	4.960	13.91%
Control	30.83	23.55	−23.61%	4.605	3.915	−14.98%

**Fig 5:**
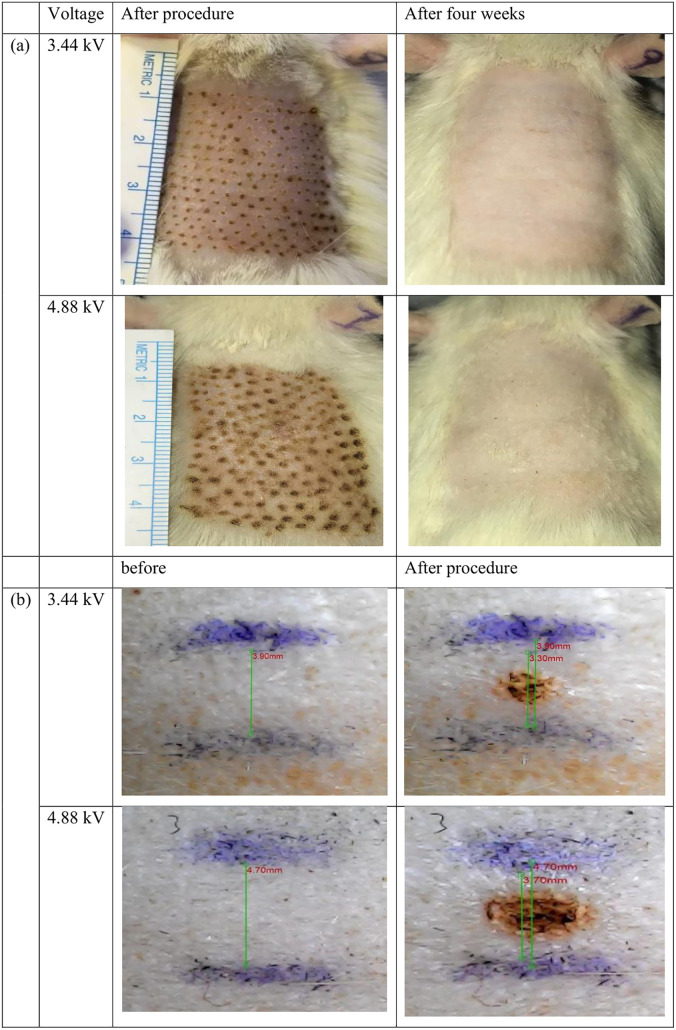
Evaluation of plasma efficacy in rats through visual inspection a) before and after four weeks. **b)** the rate of skin shrinking immediately following treatment. [16].

**Fig 6:**
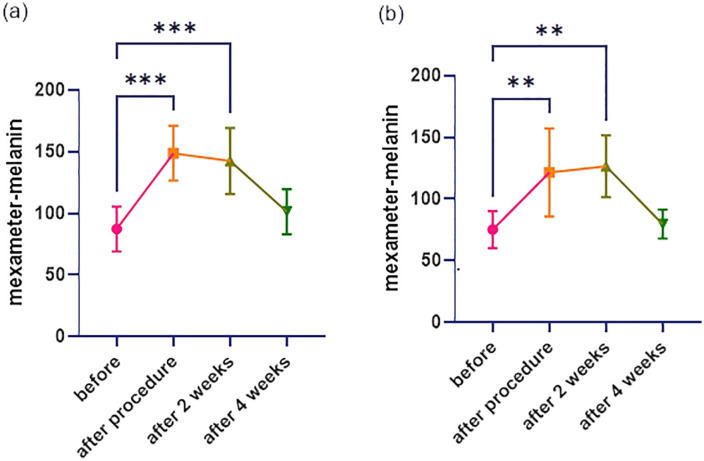
Evaluation of mouse skin mexameter parameters throughout a four-week period following plasma treatment: Melanin; (a) 4.88 kV, (b) 3.44 kV [16]. Data are presented as mean ± SEM. Statistical analysis was performed using one-way ANOVA with post-hoc test; **p < 0.01, ***p < 0.001 compared to baseline].

**Fig 7:**
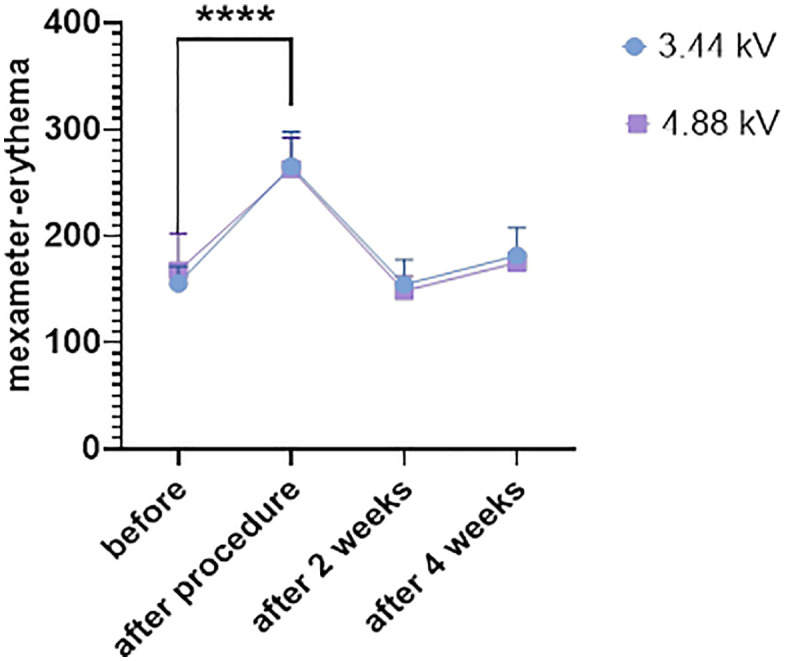
Comparison of erythema responses between 3.44 kV [16] and 4.88 kV plasma treatments over four weeks [Data are presented as mean ± SEM. Statistical analysis was performed using one-way ANOVA with post-hoc test; ****p < 0.0001 compared to baseline].

**Fig 8:**
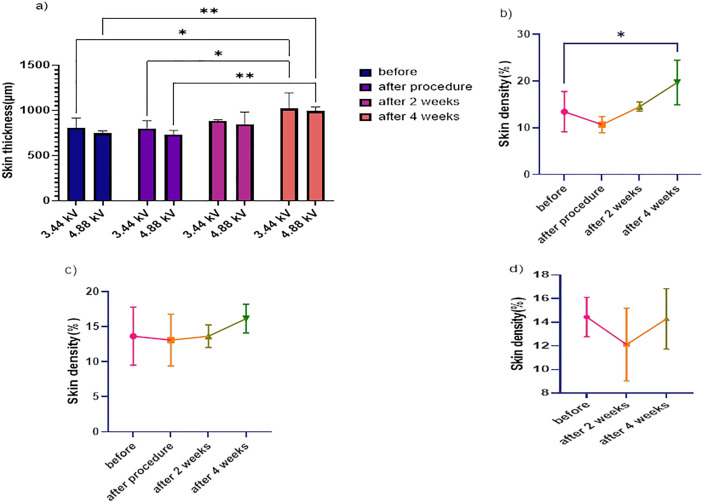
The graph illustrates the average changes in rats’ skin thickness and density over four weeks: a) Comparison of average skin thickness changes in the plasma group with a 3.44 kV voltage [16], and average skin thickness changes in the plasma group with a 4.88 kV voltage, b) Average skin density changes in the plasma group with a 3.44 kV voltage, c) Average skin density changes in the plasma group with a 4.88 kV voltage, d) Average skin density changes in the control group. [Data are presented as mean ± SEM. Statistical analysis was performed using one-way ANOVA with post-hoc test; *p < 0.05, ***p < 0.001 compared to baseline].

**Fig 9:**
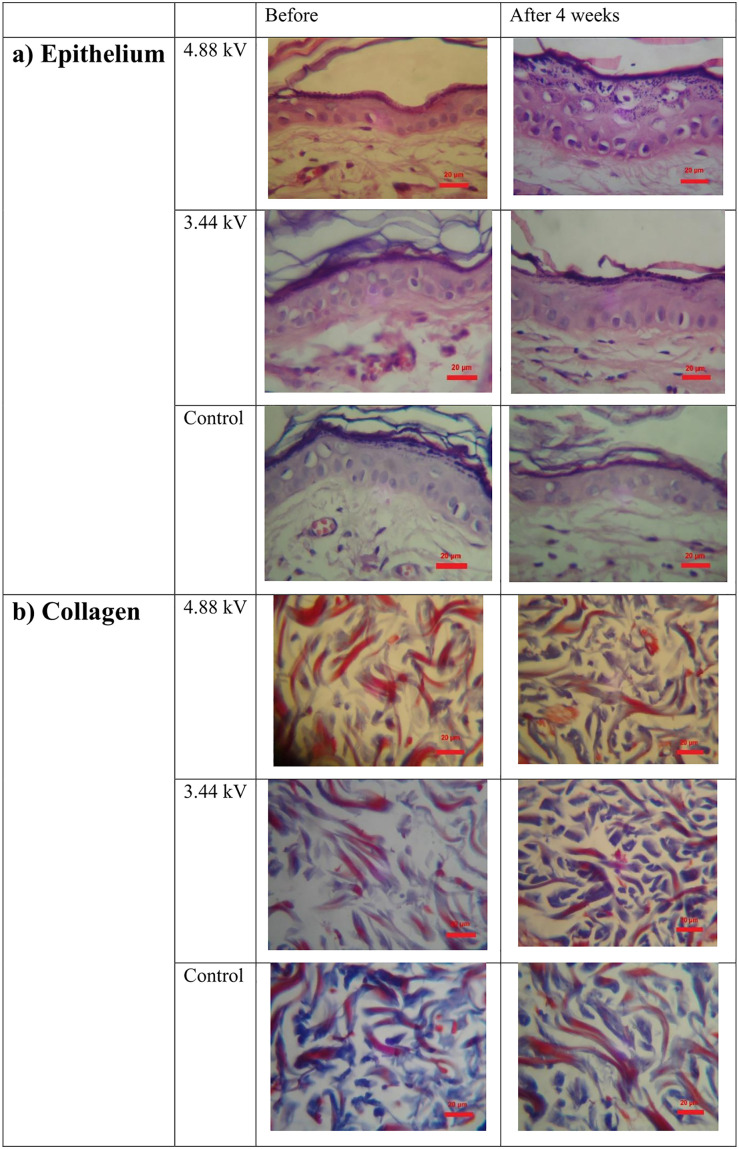
Changes in rats’ skin Histological analysis over four weeks following plasma processing (a. Epithelium and b. collagen).

### Analytical statistics

We used the same method as our earlier study [16] for our statistical analysis so that everything stays consistent and it’s easier to compare our results with previous data. We reported all our results as the average plus or minus the standard error of the mean (SEM). To check if there were differences between the three groups we tested (control, 3.44 kV, and 4.88 kV), we used one-way analysis of variance (ANOVA). When needed, we did follow-up tests to find out exactly which groups were different from each other.

All of our statistical work was done using GraphPad Prism software (version 9.0.0; GraphPad Software Inc., San Diego, CA, USA). We considered any result with a p-value lower than 0.05 to be statistically significant.

### Animal ethics and ARRIVE guidelines

Animal experiments were conducted according to the Guidelines for Animal Care and Use Committee of Tehran University of Medical Sciences. All animal experiments described were approved by the Animal Experimentation Ethics Committee of Tehran University of Medical Sciences, IRAN (IR.TUMS.MEDICINE.REC.1400.766). The in-vivo study is reported in accordance with the ARRIVE guidelines (Animal Research: Reporting of In Vivo Experiments) for reporting experiments [18,19].

## Result

### Visible evaluation of plasma performance

Fig 5. ashows how the skin looked after four weeks of plasma treatment. In the group that received 4.88 kV, the skin turned red and had some surface carbonization right after the treatment. These problems improved by the second week and were not visible anymore by the fourth week. The 3.44 kV group followed a similar pattern, but the initial changes were less noticeable.

Fig 5. bb shows how the skin’s surface length changed. In the 4.88 kV group, the average length of the treated area decreasedfrom 4.70 mm to 3.70 mm, which is a 21.27% decrease.In the 3.44 kV group, the length decreased from 3.90 mm to 3.30 mm,which is a 15.38% decrease. These results show that both groups had noticeable skin shrinkage after just one treatment.

### Influence of plasma on biophysical skin measurements

Cutometer. According to Table 2, the R2 values went up a lot and stayed high in both groups that received treatment over the four weeks. In the group that got 3.44 kV, the average R2 started at 0.5467 at the beginning, then went up to 0.6173 right after treatment, 0.6929 by the second week, and 0.7458 by the fourth week. That’s a total increase of 36.41%. For the group that got 4.88 kV, the R2 started at 0.5661, went up to 0.6369 right after treatment, then to 0.6459 in the second week, and finally to 0.7682 by the fourth week, which is a 35.7% increase from the start. These results show that skin elasticity improved over time, and both voltage levels worked about the same. On the other hand, the control group had lower R2 values, starting at 0.7265, then dropping to 0.6454 by the second week and going down further to 0.5930 by the fourth week.

**Table 2:** Comparison of skin elasticity parameters in rats after receiving plasma for four weeks [Data are presented as mean ± SEM. Statistical analysis was performed using one-way ANOVA with post-hoc test; p < 0.05 considered significant compared to baseline].

Assessment Time	Before	After operation	After 2 weeks	After 4 weeks	P value	The percentage of modifications
**R2 (control)** [16]	0.7265	–	0.6454	0.5930	p > 0.05	−18.37%
**R5 (control)** [16]	0.5842	–	0.5307	0.4986	p > 0.05	−14.65%
**R2 (3.44 kV)** [16]	0.5467	0.6173	0.6929	0.7458	0.0428 p < 0.05	36.41%
**R2 (4.88 kV)**	0.5661	0.6369	0.6459	0.7682	0.0323 p < 0.05	35.7%
**R5 (3.44 kV)** [16]	0.4627	0.6596	0.6890	0.6235	0.0258 p < 0.05	34.75%
**R5 (4.88 kV)**	0.4032	0.5936	0.6768	0.6365	0.0223 p < 0.05	57.86%

The R5 parameter showed a similar pattern. At 3.44 kV, the values went up from 0.4627 to 0.6596 right after treatment, then reached 0. 6890 in the second week, and slowly dropped to 0.6235 by week four. In the 4.88 kV group, R5 started at 0.4032, increased to 0.5936, then went up to 0.6768 in week two, and ended at 0.6365 in week four. These changes show better skin recoil and viscoelastic response in both treated groups. In the control group, R5 values went down from 0.5842 at the start to 0.5307 in the second week and further to 0.4986 by week four.

### Transepidermal Water Loss (TEWL).

As shown in Table 3, TEWL values increased immediately after treatment in both plasma-treated groups. In the 3.44 kV group, TEWL rose from 13.22 at baseline to 109.0 post-treatment, then decreased to 12.62 in week two and 10.62 by week four. In the 4.88 kV group, TEWL increased from 10.81 at baseline to 107.3 post-treatment, followed by values of 11.10 in week two and 14.16 in week four.

**Table 3:** Changes in rats’ skin TEWL parameter over four weeks following plasma processing [Data are presented as mean ± SEM. Statistical analysis was performed using one-way ANOVA with post-hoc test; *p < 0.05 considered significant].

Assessment Time	Before	After operation	After 2 weeks	After 4 weeks	P value
**3.44** kV [16]	13.22	109	12.62	10.62	0.5891 p > 0.05
**4.88** kV	10.81	107.3	11.10	14.16	0.1514 p > 0.05

### Melanin index

As shown in Fig 6, melanin levels increased markedly after treatment in both plasma groups. In the 3.44 kV group, the average value rose from 75.28 to 121.5 (Fig 6b), and in the 4.88 kV group, it increased from 87.39 to 148.8 (Fig 6a). By the fourth week of follow-up, melanin levels had decreased to 79.67 and 101.4, respectively, with values approaching baseline levels.

### Erythema index

As illustrated in Fig 7, erythema values increased immediately after treatment in both plasma groups. In the 3.44 kV group, the value increased from 167.5 to 262.9, and in the 4.88 kV group from 155.3 to 265.6. At week two, erythema levels decreased to 148.3 and 154.4, respectively. By week four, values reached 174.9 in the 3.44 kV group and 181.8 in the 4.88 kV group.

### Skin ultrasonography

As seen in Figs 8a, the average skin thickness in both groups that were treated with plasma went down right after the treatment. In the group treated with 3.44 kV, the thickness went from 810.5 μm to 801 μm. In the group treated with 4.88 kV, it dropped from 744.5 μm to 731.3 μm. By the second week, the skin thickness in both groups had gone back above the original levels and kept increasing through the fourth week. At the end of the study, the average skin thickness was 1019 μm in the 3.44 kV group, which is a 25.72% increase, and 990.7 μm in the 4.88 kV group, which is a 33.06% increase. In the control group, skin thickness went up from 852.6 μm to 908.2 μm by week two, then slightly dropped to 882.6 μm by week four, showing little change from the original level.

As shown in Fig 8b and 8c, the skin became denser in the groups that were treated with plasma. The 3.44 kV group saw an average increase in density of 46.43%, while the 4.88 kV group had an increase of 18.24%. In the control group (Fig 8d), skin density was 14.44% at the start, dropped to 12.11% after two weeks, and went back up to 14.29% by week four.

### Histological analysis

As shown in Fig 9 and Table 4, histological analysis revealed marked differences in both epidermal and collagen thickness between groups. In the 4.88 kV group, average epithelial thickness increased from 26.46 μm to 43.86 μm, representing a 65.75% increase. In the 3.44 kV group, the increase was from 32.84 μm to 35.86 μm (9.19%). Conversely, the control group showed a reduction in epithelial thickness from 30.83 μm to 23.55 μm, corresponding to a 23.61% decrease. Regarding collagen measurements, the 4.88 kV group demonstrated an increase from 4.015 μm to 4.685 μm (16.68%), and the 3.44 kV group from 4.270 μm to 4.960 μm (13.91%). The control group showed a decrease from 4.605 μm to 3.915 μm, equivalent to a 14.98% reduction.

Hair follicles are normally found in the back skin of rats, but all measurements were taken from areas that had been shaved to avoid any interference. The increase in skin thickness was confirmed through microscopic examination, showing that both the outer and inner layers of the skin had grown thicker, regardless of how the hair follicles were developing. Even though hair follicles were present in the full skin sections, the study focused on the areas between the follicles. The effects of the treatment on the hair follicles themselves were not studied in this research.

## Discussion

As the global population ages, the demand for non-invasive methods to restore skin structure and function continues to grow. Aging skin undergoes progressive deterioration, including reduced elasticity, thinning, uneven pigmentation, and increased wrinkling—changes driven by intrinsic (genetic and metabolic) and extrinsic (UV exposure, oxidative stress) factors [20–22]. In recent years, Cold Atmospheric Plasma (CAP) has emerged as a promising non-invasive modality for dermatologic rejuvenation due to its multifaceted effects on tissue remodeling, cellular signaling, and wound healing [16,17,23].

In this study, spark plasma—a form of CAP—was applied at two voltage levels (3.44 kV and 4.88 kV) to assess its effect on key biophysical and structural parameters of rat skin. Across all measured variables, both voltages induced improvements, though the higher voltage (4.88 kV) consistently produced greater effects, indicating a voltage-dependent biological response.

Skin elasticity, a central indicator of dermal health, showed measurable improvements through R2 and R5 parameters, increasing by 36.41% and 48.18% in the 3.44 kV group, and by 35.7% and 57.86% in the 4.88 kV group, respectively. These changes are consistent with enhanced viscoelastic behavior and align with previous CAP studies demonstrating fibroblast stimulation and extracellular matrix remodeling [16,17].

TEWL values rose sharply immediately after treatment (up to 109 g/h/m²), reflecting initial thermal impact on the stratum corneum. However, by week four, values had returned to baseline or lower (e.g., 10.62 in the 3.44 kV group), supporting the concept of full skin barrier recovery and re-establishment of hydration regulation—an effect previously described as “protective layer reformation” in CAP-related literature [14,24].

Melanin and erythema indices also followed expected temporal patterns. Both increased immediately post-treatment, likely due to superficial inflammation and reactive melanogenesis. However, their decline to near-baseline by week four (e.g., melanin returning to 79.67 in 3.44 kV and 101.4 in 4.88 kV) suggests transient rather than persistent pigmentary or inflammatory effects. This supports earlier findings that CAP does not require chromophore interaction and does not provoke long-term hyperpigmentation [17,25].

Ultrasound scans showed that the tissue had two different reactions. At first, the skin became thinner, like from 810.5 to 801 micrometers when using 3.44 kV. But by the fourth week, the skin got thicker again, even more than it was before. For example, with 3.44 kV, the skin was up to 1019 micrometers thick, and with 4.88 kV, it was 990.7 micrometers. This means the skin increased in thickness by 25.72% and 33.06% respectively, which shows the skin is rebuilding and the dermis is getting thicker. At the same time, the skin became denser, with density values rising up to 46.43% in the 3.44 kV group. This suggests the skin is becoming more compact and has more collagen. Looking at the skin under a microscope also showed that the outer layer, called the epithelium, got thicker by 65.75% in the 4.88 kV group, and the collagen fibers expanded by up to 16.68%. In contrast, the control group saw a decrease in these measurements. These findings strongly show that the tissue is healing and making new extracellular matrix, which is probably because fibroblast cells are getting activated and there’s less oxidative stress involved [16,23,26].

When comparing the two voltage levels, it was found that both helped improve all the measured factors. However, the higher voltage (4.88 kV) made a bigger difference in how collagen was formed and how the epithelial tissue was rebuilt. This suggests that higher voltage may have a stronger effect, which could be because more reactive species are created [20], or the heat goes deeper into the tissue [17]. Importantly, these improvements didn’t cause ongoing inflammation or problems with the tissue barrier, which shows that spark plasma treatment is safe when used properly.

Compared to traditional laser treatments that remove layers of skin, spark plasma has some good benefits. These include not needing specific pigments to work, less time needed for recovery, and a lower chance of darkening of the skin after inflammation [27–33]. The results from this study support the idea that cold atmospheric plasma can be a good alternative to lasers for improving skin appearance. This matches other reports that talk about how plasma can help skin heal faster, make it more hydrated, and improve the structure of collagen [16,17,26].

In conclusion, this study demonstrates that a single session of spark plasma treatment significantly improves skin elasticity, thickness, density, and histological architecture in a voltage-dependent manner. The results support the clinical potential of spark plasma as a non-invasive strategy for skin rejuvenation, particularly in addressing features such as laxity, atrophy, and barrier weakening. Further investigations should evaluate long-term effects, optimize treatment protocols, and assess translational applicability in human skin models.

## Conclusion

This study demonstrates that spark plasma therapy—an emerging Cold Atmospheric Plasma (CAP) modality—can induce measurable improvements in key biophysical and structural parameters of the skin, including elasticity (R2, R5), transepidermal water loss (TEWL), melanin and erythema indices, thickness, and collagen content [14,16,17]. Treatments at both 3.44 kV and 4.88 kV produced positive effects, with more pronounced outcomes observed at the higher voltage level, particularly in terms of epithelial thickening and collagen remodeling [16,26].

These findings expand upon earlier work by our group [16], offering new quantitative insights into voltage-dependent biological responses to plasma exposure. The results suggest that spark plasma may serve as a safe, non-invasive, and tunable method for promoting skin rejuvenation [17,23]. Additionally, the differential effects observed between voltage levels may help guide the development of optimized treatment protocols tailored to specific clinical indications. Future research should focus on long-term safety, repeated exposures[24,25], and translational validation in human skin models.

## Supporting information

S1 Histological. (XLSX)

S2 Mexameter, tewameter. (XLSX)

S3 Cutometer, sonography. (XLSX)
